# Hepatic steatosis in postmenopausal women is characterized by distinct serum extracellular vesicle proteomic signatures

**DOI:** 10.1186/s12916-025-04571-4

**Published:** 2025-12-07

**Authors:** Patrick Pirrotte, Brooke Lovell, Siobán D. Harlow, Carrie A. Karvonen-Gutierrez, Michelle M. Hood, Ignazio S. Piras, Xiumei Wu, Melissa N. Martinez, Ritin Sharma, Krystine Garcia-Mansfield, Maya Willey, Johanna K. DiStefano

**Affiliations:** 1 Division of Early Detection and Prevention, Translational Genomics Research Institute, Phoenix, AZ USA; 2 Integrated Mass Spectrometry Shared Resource, City of Hope Comprehensive Cancer Center, Duarte, CA USA; 3 Department of Epidemiology, School of Public Health, University of Michigan, Ann Arbor, MI USA; 4 Metabolism Research Unit, Translational Genomics Research Institute, Phoenix, AZ USA

**Keywords:** Liver, Hepatic steatosis, Proteomics, Extracellular vesicles, Midlife, MASLD

## Abstract

**Background:**

Metabolic dysfunction-associated steatotic liver disease (MASLD) is common among midlife women. Circulating extracellular vesicles (EVs) carry bioactive cargo that may mediate or reflect disease processes, but their role in hepatic steatosis in postmenopausal women remains unexplored.

**Methods:**

We conducted liquid chromatography data-independent acquisition–mass spectrometry on serum-derived EVs from 275 postmenopausal women enrolled in the Michigan site of the Study of Women’s Health Across the Nation (MI-SWAN). Participants were grouped by hepatic steatosis status (*n* = 75), assessed via standardized ultrasound at the 2010 follow-up visit. Fasting serum samples were processed using size exclusion chromatography to isolate EVs. Differential EV protein abundance was evaluated by ANCOVA, adjusting for ethnicity and diabetes status, and applying Benjamini–Hochberg correction. Gene Set Enrichment Analysis (GSEA) was performed to identify enriched biological pathways.

**Results:**

Among 469 detected EV proteins, 60 differed by hepatic steatosis status (*p* < 0.05), with two proteins remaining significant after multiple testing correction: complement C4A (C4A) and afamin (AFM). GSEA indicated enrichment in lipid metabolism and innate immune activation pathways. Subgroup analyses revealed racial and disease severity-specific differences in EV protein profiles. In Black women (*n* = 172), AFM, C4A, and APOA1 were significantly elevated, while in White participants (*n* = 103), no proteins reached significance, although AFM displayed a nonsignificant trend toward higher abundance. In participants with severe hepatic steatosis (*n* = 43), subgroup analysis showed increased COL18A1, AFM, PRG4, and INHBE and decreased C4A and APOA1. INHBE was the only protein consistently elevated across all three subgroups, whereas others showed subgroup-specific enrichment, such as immunoglobulins in Black women and complement or coagulation proteins in White participants and those with severe steatosis. Analysis of hepatic transcriptomic datasets demonstrated consistently higher *INHBE* expression across the MASLD spectrum, including metabolic dysfunction-associated steatohepatitis (MASH), while *AFM* expression was significantly higher in the MASH vs. steatosis comparison.

**Conclusions:**

This study demonstrates that circulating EV proteomes differ by hepatic steatosis status in postmenopausal women. While exploratory, candidate EV proteins such as INHBE and AFM merit validation as biomarkers and potential contributors to MASLD in this high-risk population.

**Supplementary Information:**

The online version contains supplementary material available at 10.1186/s12916-025-04571-4.

## Background

With increasing longevity, women now spend nearly 40% of their lives post-menopause [1]. By 2030, postmenopausal women will comprise nearly half of the female population in the USA [2], yet the metabolic consequences of this transition remain incompletely defined. The reduction in estrogen production triggers profound shifts in fat distribution, insulin sensitivity, and dyslipidemia, all of which are associated with metabolic dysfunction-associated steatotic liver disease (MASLD) [3–10]. The biological mechanisms linking hormonal decline to metabolic dysfunction represent an underexplored area of research. As the number of postmenopausal women continues to rise, there is a pressing need to disentangle the hormonal drivers of metabolic dysfunction from other risk factors to inform targeted prevention and treatment strategies.

MASLD is a chronic condition defined by excessive triglyceride accumulation in the liver, spanning a disease spectrum from isolated steatosis, where at least 5% of liver weight consists of intracellular fat, to metabolic dysfunction-associated steatohepatitis (MASH), which involves liver inflammation and hepatocellular injury [11]. While MASLD has historically been more prevalent in men, its burden among women has risen sharply in recent decades, increasing from 18.5% in 1988–1994 to 24.9% in 2007–2014 [12]. This increase has outpaced that seen in men [13] and has been accompanied by a higher mortality rate in affected women [14]. According to one study, postmenopausal women showed a nearly two-fold higher likelihood of advanced fibrosis (36.1%) compared to men (17.7%) [15]. Furthermore, MASH is currently the leading indication for liver transplantation in women [16]. Animal studies provide evidence that estrogen loss directly increases susceptibility to hepatic steatosis and steatohepatitis, particularly in the presence of mild metabolic dysfunction, such as moderate adiposity, dyslipidemia, or prediabetes [4]. Despite these trends, our understanding of the mechanisms by which MASLD develops and progresses in postmenopausal women is limited.

Routine clinical assessments of liver health have well-recognized limitations in detecting early or heterogeneous stages of MASLD [17, 18]. Serum alanine aminotransferase (ALT) and aspartate aminotransferase (AST) lack both sensitivity and specificity, with up to 70% of individuals with MASLD exhibiting normal enzyme levels [19]. Abdominal ultrasound, although non-invasive and widely available, typically requires ≥ 20–30% hepatic fat content for reliable detection and shows reduced sensitivity in individuals with mild steatosis or higher body mass index [20–23]. Non-invasive fibrosis scores (e.g., FIB-4, NAFLD fibrosis score) effectively exclude advanced fibrosis but have limited precision in intermediate-risk groups and cannot resolve early disease [24]. Together, these limitations hinder early detection and risk stratification in postmenopausal women, a population in which timely identification of hepatic dysfunction could inform prevention and therapeutic strategies.

Extracellular vesicles (EVs) are small membrane-bound vesicles released by cells that mediate intercellular communication through the transfer of bioactive molecules [25, 26]. Because they carry cargo that mirrors the metabolic or pathological state of their cell of origin, EVs provide insight into underlying cellular function [27]. Growing evidence suggests that EVs contribute to metabolic dysfunction by transporting signaling molecules that influence diverse pathological processes, including MASLD [28–34]. Circulating EV levels are elevated in individuals with MASLD and correlate positively with disease severity [33, 35, 36]. EVs also modulate key hepatic processes, including intracellular signaling, tissue injury and repair, and matrix remodeling [37]. In animal models, EVs released from steatotic hepatocytes alter the metabolome, reprogram hepatic gene expression, and exacerbate liver fibrosis and inflammation. For example, in mice fed a diet high in saturated fat, cholesterol, and fructose, increased levels of hepatocyte-specific EVs in the circulation tracked with disease progression, whereas inhibition of EV release attenuated hepatic injury [38]. These findings suggest that hepatocyte-derived EVs actively drive disease progression rather than merely reflect liver injury, supporting their potential relevance for therapeutic targeting and translational studies.

Beyond their mechanistic role in disease progression, EVs are emerging as promising biomarkers for early disease detection. Unlike conventional methods that typically focus on a single type of molecule, EVs carry a diverse array of biomolecules, including proteins, DNA, lipids, and RNA, offering a more comprehensive molecular context [39]. This cargo is protected by a lipid bilayer, which confers stability and retains the cell-type-specific molecular signatures of the originating cell. Because EVs circulate in the blood, they can be sampled using minimally invasive techniques [40–42]. As a result, EVs carrying disease-specific markers have been observed to circulate long before clinical symptoms appear, demonstrating potential utility for identifying subclinical changes [39, 43, 44]. Importantly, the isolation of disease-specific EVs improves assay sensitivity by allowing for the removal of highly abundant, non-specific proteins that can mask low-abundance biomarkers [45]. By capturing integrated molecular information from both hepatic and systemic processes, EVs have the potential to complement existing diagnostic tools and yield mechanistic insights into the molecular pathways driving the full spectrum of MASLD.

Although EV research in MASLD is progressing, studies in postmenopausal women are scarce [46–50]. To address this gap, we leveraged clinical data and serum samples from the Study of Women’s Health Across the Nation (SWAN), a multi-site, longitudinal cohort study designed to investigate the health of women throughout midlife and the menopausal transition [51]. In 2010, the Michigan site (MI-SWAN) conducted liver ultrasounds to assess hepatic steatosis among predominantly postmenopausal participants [52], with corresponding serum samples archived in the MI-SWAN biorepository. This resource provides a unique opportunity to characterize EV-derived proteomic profiles in a diverse postmenopausal population with hepatic steatosis, enabling the identification of candidate biomarkers and insights into disease mechanisms.

Here, we sought to identify and characterize EV-derived proteomic profiles associated with abdominal ultrasound-determined hepatic steatosis in postmenopausal women, leveraging the comprehensive clinical characterization of MI-SWAN participants to deepen understanding of EVs as potential biomarkers and therapeutic targets for MASLD.

## Methods

### Study participants

SWAN is an ongoing community-based cohort study involving multiple racial and ethnic groups, designed to characterize the menopausal transition, post-menopause, and related health changes. Women aged 42–52 years were recruited from defined sampling frames at seven clinical sites in 1996–1997 (study design details are available elsewhere [51]). To be eligible, participants were required to have an intact uterus and at least one ovary, and to have experienced a menstrual period within the preceding 3 months. Exclusion criteria included hormone therapy use in the prior 3 months, pregnancy, or breastfeeding. Participants subsequently completed up to 17 follow-up visits. All participants provided informed consent, and study procedures were approved by the institutional review board at each clinical site.

The MI-SWAN recruited 543 women, with participants self-identifying as Black (66%) or White (34%). Participants were classified as postmenopausal based on the absence of menses for at least 12 consecutive months. At the 2010 annual follow-up visit, hepatic steatosis was assessed using ultrasound imaging [52]. Of the 403 women who participated in that visit, 345 (86%) underwent hepatic ultrasound. Participants with a history of cirrhosis or chronic liver disease attributable to viral hepatitis or hemochromatosis (*n* = 14) were excluded, resulting in 331 potentially eligible participants. Stored serum samples were available for 278 of these participants and three women who were not postmenopausal at the time were excluded. Thus, 275 women comprised the analytical sample set for this study.

At each study visit, women completed questionnaires providing information on sociodemographic characteristics, menopausal status, health status, and medications. Anthropometric measurements including height, weight, and waist and hip circumferences, as well as a fasting blood draw, were obtained. Diabetes was defined as the use of glucose-lowering medication during the study, fasting glucose ≥ 126 mg/dL at two consecutive visits, or self-reported diabetes at two visits with fasting glucose ≥ 126 mg/dL at one visit. Blood was refrigerated for 1–2 h prior to centrifugation, and serum was aliquoted, frozen, and stored at − 80 °C.

### Abdominal ultrasound for hepatic fat evaluation

A single ultrasound technician, blinded to the medical history of participants, performed abdominal ultrasounds using a Sonoline Elegra Ultrasound Imaging System (Siemens Medical Systems Inc.) equipped with a 3.5 MHz transducer and a 411 LE 0.5 phantom (GAMMEX-RMI Ltd). All images were evaluated for markers of hepatic steatosis by a single radiologist applying a standardized protocol and blinded to participant profile, as previously reported [52]. The liver was graded for markers of hepatic steatosis including bright hepatic echo pattern compared with echo response of the right kidney, attenuation of the echo beam, and presence of focal fatty sparing [53]. Hepatic steatosis was categorized as “moderate/severe” or “none/mild” based on echogenicity and clarity of structures within the liver [54].

### Isolation of EVs from serum

EVs were isolated by size exclusion chromatography using qEVoriginal 70 nm Gen 2 columns (Izon Science; Medford, MA) from fasting serum samples. Following column equilibration with 15 mL of phosphate buffered saline (PBS), 500 µL of serum was loaded onto the column and 6 × 500 µL fractions (F) were collected (F7–F12), following the collection of the 3 mL void volume. Fractions F7–F12 were combined and concentrated with 50 K Amicon filters (Millipore, UFC805096) to a final volume of 500 µL. The combined concentrated EVs were used for downstream analyses.

### Sample preparation for proteomics analysis

Isolated EVs were solubilized in a 2% sodium deoxycholate-based lysis buffer and sonicated using a cup-horn shaped sonotrode (UTR2000, Hielscher Ultrasonics) for 30 s (15 s on 1 s off) at 50% amplitude for three rounds of sonication. Protein extracts were clarified by centrifugation and protein concentration was determined using the BCA assay (Pierce). Proteins (40 µg) from isolated EVs were processed as previously described [34]. To generate a reference spectral library, equal amounts of protein (i.e., 5 µg) were combined from each sample to create a 1.39 mg pool. The pooled sample was processed as described [34]. Peptides were subjected to offline fractionation via high pH reverse phase chromatography on an Ultimate 3000 HPLC system (Thermo Scientific; Waltham, MA). Peptides were loaded onto a 10 cm C18 column (Waters XBridge C18, 4.6 mm ID, 3.5 µm particle size) and eluted over a 96-min method into a 96-well plate. The resulting 96 fractions were combined to 24 peptide fractions for LC–MS/MS acquisition. All samples and library fractions were spiked with iRT peptides (Biognosys; Switzerland).

### Liquid chromatography–data-independent acquisition mass spectrometry (LC-DIA/MS)

All mass spectrometry data were acquired on a nanoElute liquid chromatography system coupled to a timsTOF HT Pro 2 (Bruker Daltonics; Billerica, MA) mass spectrometer with a captive spray source (Bruker) using a 62-min LC gradient at a flowrate of 850 nL/min on a 25 cm C18 column (Bruker PepSep, 150 µm ID, 1.5 µm particle size). Individual library fractions were acquired in DDA-PASEF (data-dependent acquisition–parallel accumulation serial fragmentation) mode with MS1 scans covering a mass range of 100–1700 m/z, TIMS mobility window (1/K0) between 0.70 and 1.50 with 75 ms accumulation and ramp time. DDA scans involved 7 PASEF ramps for a total cycle time of 0.65 s and a collision energy ramp of 20 to 65 eV for ion mobility window (1/K0) of 0.6–1.6. Each EV sample was acquired in DIA (data-independent acquisition)-PASEF mode keeping the same accumulation and ramp time as DDA runs. The capillary voltage was kept at 1700 V and dry gas temperature was kept at 200 °C.

### Proteomics data analysis

Spectral libraries from the DDA-PASEF runs were created with Spectronaut 19.8 software against a human SwissProt database (UP000005640, downloaded June 2025). Theoretical digestion was performed using trypsin allowing for a maximum of two missed cleavages. Cysteine carbamidomethylation was set as a fixed modification, while methionine oxidation and protein N-terminal acetylation were set as variable modifications. PSMs and peptides were filtered for false discovery rate (FDR) < 1%. DIA-MS data was searched against data-specific spectral libraries using default parameters (cross-run normalization, data imputation, and scaling were disabled). Protein abundances were normalized using variance stabilization normalization (*vsn* package) [55]. To determine the amount of variation in protein abundance explained by clinical variability, principal component analysis (PCA) from *PCAtools* was run on proteins present in 100% of samples and clinical variables reporting the first 25 principal components (PCs). The significance of the relationship between confounding factors and protein abundance was calculated via *eigencorplot* function on variables for the first five PCs. Differential abundance was calculated using an ANCOVA with a Benjamini–Hochberg correction for multiple testing, accounting for ethnicity and diabetes status. Significant proteins (*p* < 0.05) were filtered for presence in at least 50% of samples and partial least squares discriminant analysis (PLS-DA) was used to assess group separation at 95% confidence with the *plsda* function in the *mixOmics* package [56]. A Hotelling’s *t*-squared statistic was calculated using the *Hotelling* package to determine significance of group separation (*p* < 0.05). A significance score, defined as − log_10_(*p* value) × sign(log_2_FC), was calculated for all identified EV proteins. This score was used to order proteins based on statistical significance and fold-change direction for subsequent Gene Set Enrichment Analysis (GSEA). Analysis was performed in GSEA software v 4.4.0 [57] against the Gene Ontology Biological Processes (GOBP) database (v2025).

## Results

### Characteristics of the study cohort

Table 1 summarizes demographic and clinical characteristics of the 275 postmenopausal participants at the 2010 follow-up visit. Participants with hepatic steatosis (*n* = 75) had higher BMI, greater waist circumference, and a higher prevalence of T2D compared to those without steatosis (*n* = 200). They also exhibited elevated triglyceride (TG), fasting glucose, and HbA1c levels. Among participants with hepatic steatosis, those with T2D had greater adiposity and more pronounced metabolic dysfunction, including higher TG, fasting glucose, and HbA1c levels (Additional file 1: Table S1).

**Table 1:** Study cohort demographic information and clinical characteristics (*N* = 275)

Parameter	Hepatic steatosis	No hepatic steatosis
***N***	75	200
Age (y)	59.48 ± 2.89	58.89 ± 2.76
Race ***n*** (%)		
Black	38 (50.7)	134 (67.0)
White	37 (49.3)	66 (33.0)
BMI (kg/m^2^)	37.2 ± 7.6	32.7 ± 8.1
T2D, ***N*** (%)	32 (42.7)	47 (23.5)
WC (cm)	109.6 ± 13.1	98.0 ± 16.4
TG (mg/dL)	153.8 ± 90.7	109.5 ± 51.3
FG (mg/dL)	128.1 ± 76.1	96.9 ± 31.1
HbA1c (%)	6.8 ± 1.8	6.0 ± 0.8

Data are means ± standard deviation, unless otherwise indicated; *WC *waist circumference, *TG *fasting serum triglyceride levels, *FG *fasting plasma glucose, *HbA1c *glycosylated hemoglobin

### Proteomic profiling of serum EVs

Label-free LC-DIA/MS proteomic analysis of serum-derived EVs identified 469 proteins across the study cohort. To assess potential confounding effects of clinical variables on protein abundance, PCA was performed using proteins detected in all samples, with clinical covariates evaluated for their contribution to variance. This analysis indicated that TG levels and ethnicity could potentially influence protein profiles (Additional file 1: Fig. S1). Consequently, subsequent differential abundance analyses were adjusted for ethnicity, as well as T2D status, given their established contributions to MASLD risk independent of lipid metabolism. TG levels were not included as covariates, as they reflect a pathological feature of MASLD rather than an independent confounding variable [58].

### Differential abundance of EV proteins in hepatic steatosis

**Fig. 1:**
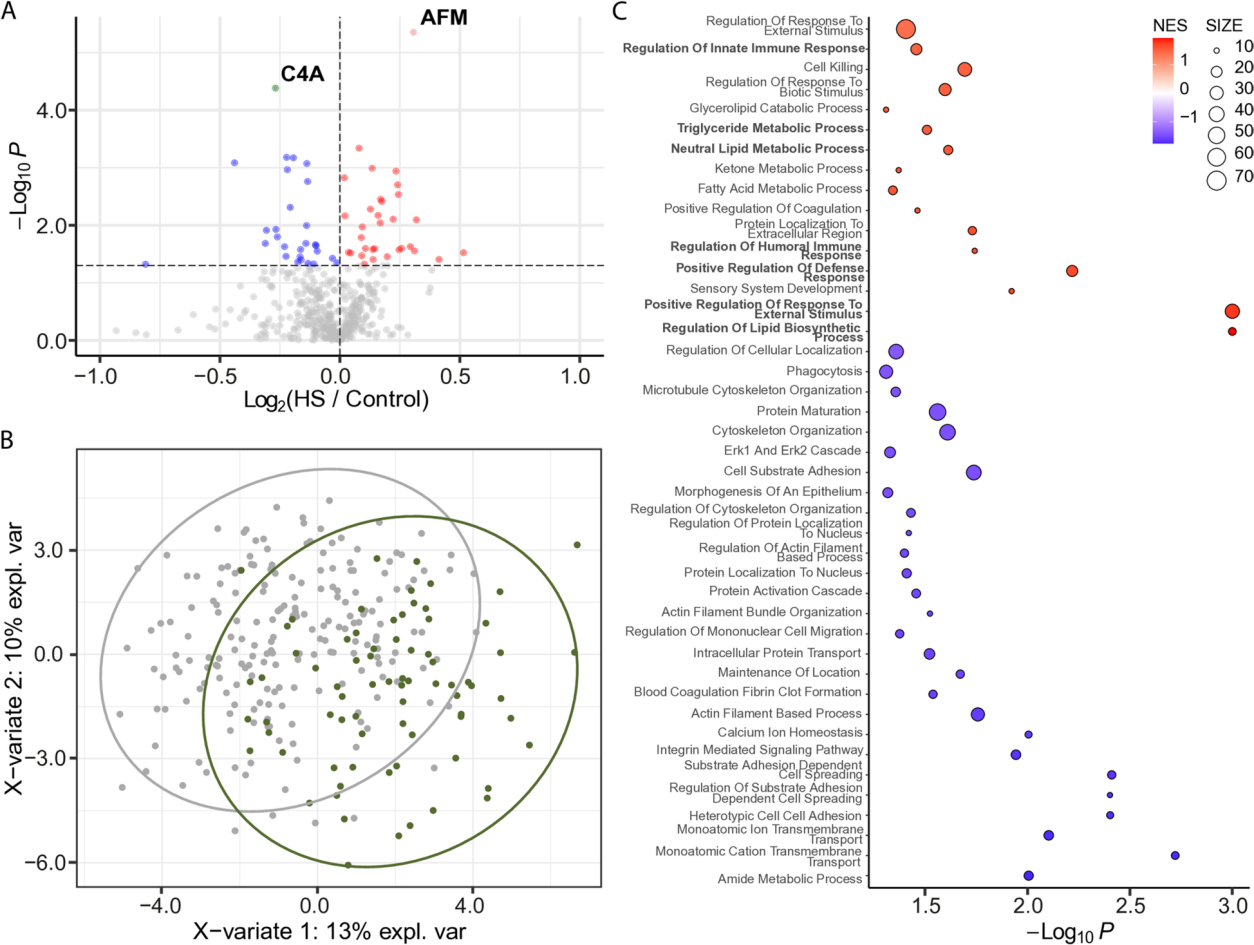
Differentially abundant EV proteins in participants with hepatic steatosis (HS) vs. controls. **A** Volcano plot displaying differentially abundant proteins. The horizontal dotted line indicates − log_10_ (0.05). Red and pink dots represent proteins significantly increased in hepatic steatosis (*p* < 0.05 and FC > 0; *q* < 0.05 and FC > 0). Blue and green dots represent proteins significantly decreased (*p* < 0.05 and FC < 0; *q* < 0.05 and FC < 0). Gray dots are not significant. **B** Partial least squares discriminant analysis (PLS-DA) plot showing separation between participants with hepatic steatosis (green circles) and controls (gray circles). Proteins were filtered for *p* < 0.05 and presence in ≥ 50% of samples. Ovals represent 95% confidence intervals. **C** Dot plot showing significantly enriched Gene Ontology (GO) Biological Processes. Dots represent gene sets and are colored by normalized enrichment score (NES); dot size reflects the number of genes in each set

We identified 60 differentially abundant proteins (DAPs) between participants with hepatic steatosis and controls (*p* < 0.05; Fig. 1A). Partial least squares discriminant analysis (PLS-DA) of significant proteins (*p* < 0.05) detected in at least 50% of samples demonstrated clear separation between samples groups (Fig. 1B), which was further confirmed by Hotelling’s *T*^2^ test (*p* < 0.05). After correcting for multiple comparisons, two proteins remained significant (*q* < 0.05): C4A (complement factor 4A), which was decreased in hepatic steatosis, and afamin (AFM), which was increased (Fig. 1A).

We performed GSEA (GO Biological Processes) to gain functional insights into protein cargoes carried by EVs from participants with hepatic steatosis (Fig. 1C). Significant and positively enriched gene sets (*p* < 0.05) were generally involved in lipid biology (i.e., triglyceride metabolic process, neutral lipid metabolic process, regulation of lipid biosynthesis process) and inflammation and immunity (e.g., regulation of innate immune response, regulation of humoral immune response, positive regulation of defense response, and positive regulation of response to external stimulus).

### EV proteins differ between Black and non-Hispanic White participants with steatosis

**Fig. 2:**
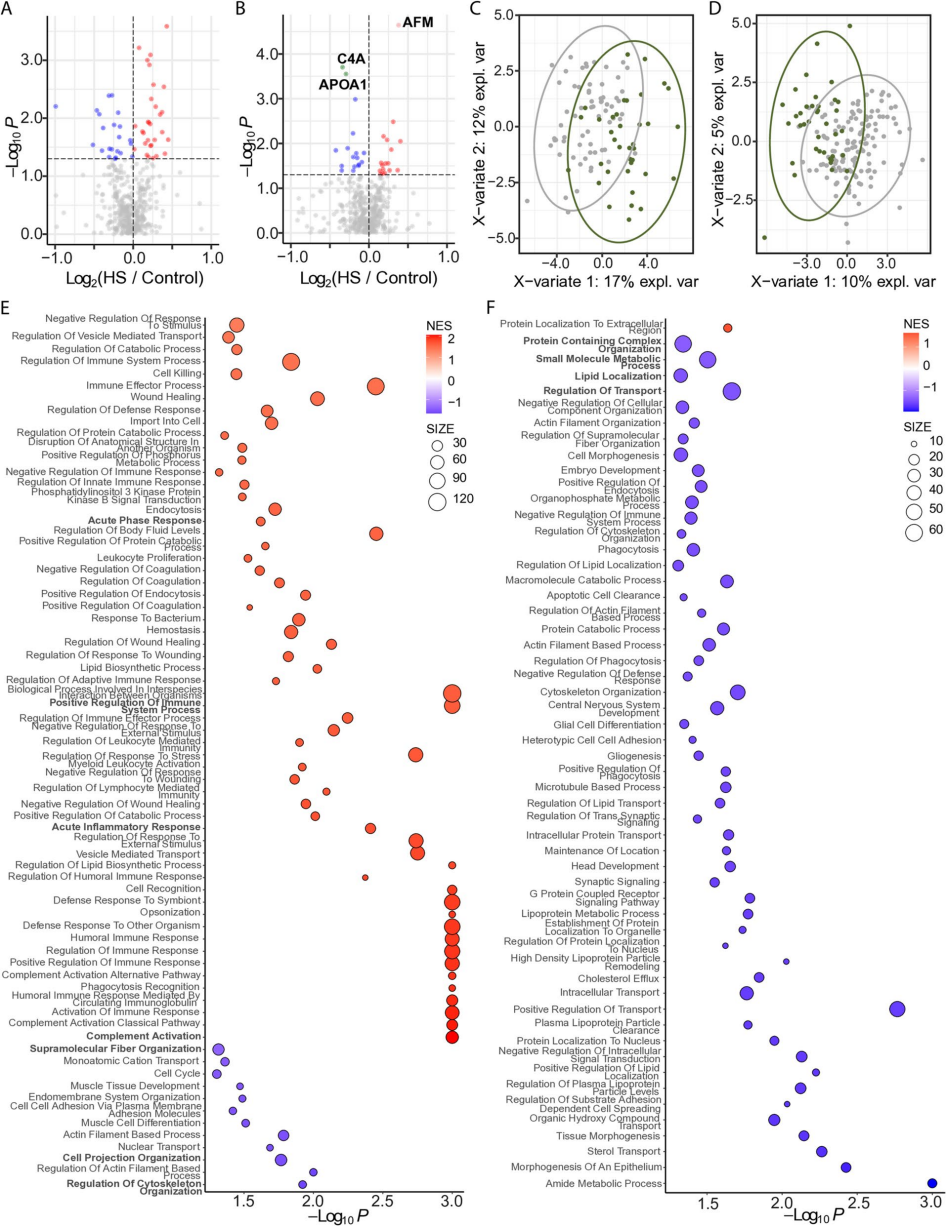
EV proteomic differences by race. Volcano plots displaying differentially abundant proteins within the White (**A**) and Black (**B**) subgroups. The horizontal dotted line indicates − log_10_ (0.05). Red and pink dots represent proteins significantly increased in hepatic steatosis (*p* < 0.05 and FC > 0; *q* < 0.05 and FC > 0). Blue dots indicate proteins significantly decreased (*p* < 0.05 and FC < 0). Gray dots are not significant. PLS-DA plots showing separation of hepatic steatosis (green circles) and controls (gray circles) within the White (**C**) and Black (**D**) subgroups. Proteins were filtered for *p* < 0.05 and presence in ≥ 50% of samples. Ovals represent 95% confidence intervals. Dot plots showing significantly enriched GO Biological Processes in the White (**E**) and Black (**F**) cohorts. Dots represent gene sets and are colored by NES; dot size reflects the number of genes in each set. HS, hepatic steatosis

Given our previous observation of a higher prevalence of hepatic steatosis among non-Hispanic White participants compared to Black participants in this cohort [52], we further examined the effect of steatosis by race/ethnicity. In White participants (*n* = 103), differential abundance analysis identified 52 DAPs (*p* < 0.05) after adjusting for T2D status; however, none remained statistically significant following multiple testing correction (Fig. 2A). In Black women (*n* = 172), 34 DAPs (*p* < 0.05) were detected; three of which, AFM, C4A, and APOA1, remained significantly different following multiple testing correction (Fig. 2B). Although AFM levels were not significantly altered in White participants, a trend toward higher abundance was observed (*p* = 0.138). PLS-DA showed clear distinction between the control and the hepatic steatosis groups in both comparisons (Fig. 2C and D). Hotelling’s *T*^2^ testing showed significant separation between groups (*p* < 0.05).

GSEA revealed additional distinctions between serum EV proteins from White and Black participants with hepatic steatosis. In White participants, positively enriched processes (*p* < 0.05) were broadly involved in innate and humoral responses, such as acute phase response, positive regulation of immune system process, complement activation pathway, and acute inflammatory response, while negatively enriched processes included supramolecular fiber organization, cell projection organization, and similar cytoskeleton organization processes (Fig. 2E). In Black participants, processes related to protein containing complex organization, small molecule metabolic process, lipid localization, and regulation of transport were negatively enriched (Fig. 2F).

### Participants with severe steatosis exhibit EV proteins associated with lipid transport dysregulation, extracellular matrix remodeling, and inflammation

**Fig. 3:**
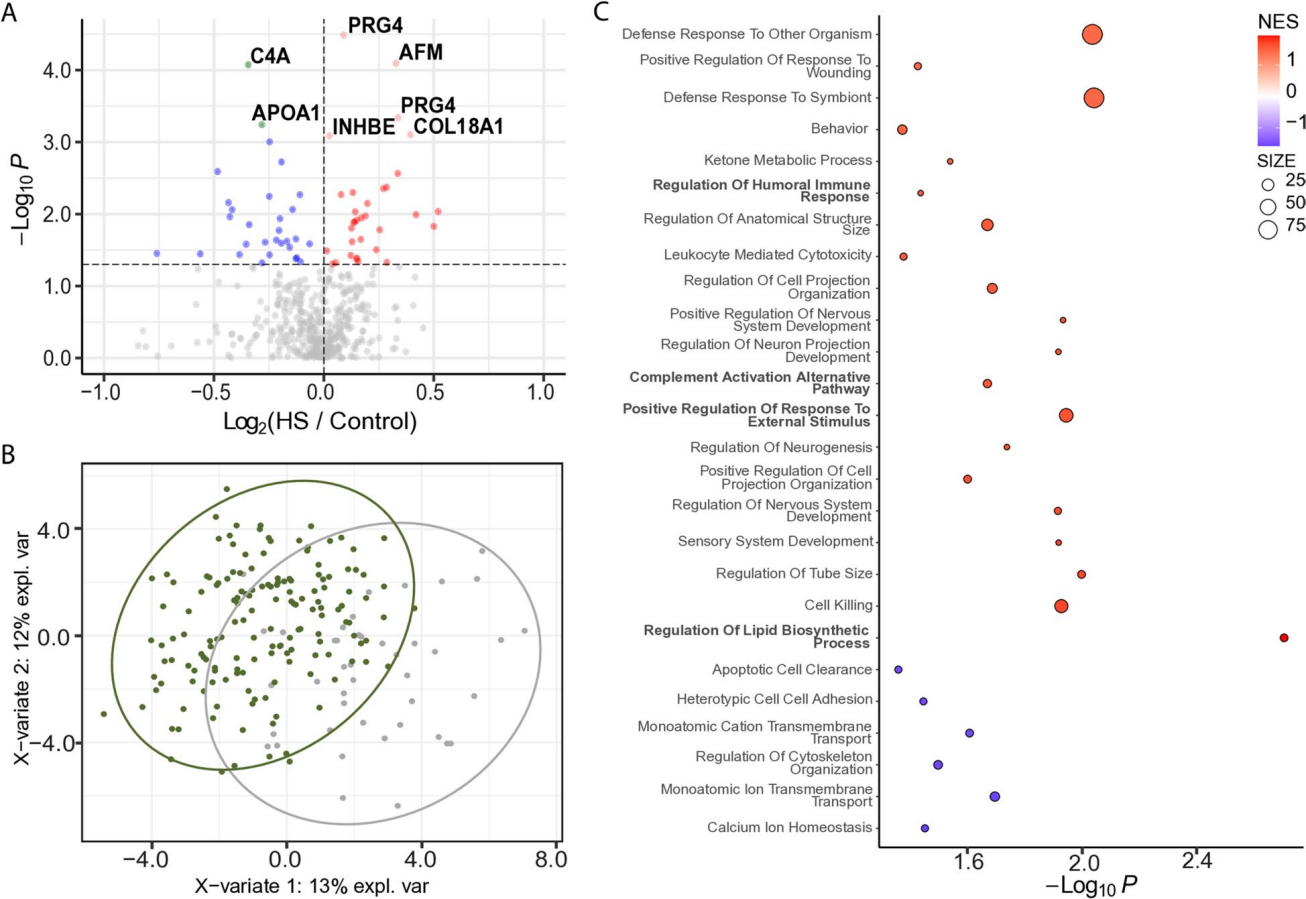
EV protein signatures in participants with severe hepatic steatosis (HS). **A** Volcano plot displaying differentially abundant proteins in participants with severe hepatic steatosis. The horizontal dotted line indicates − log_10_ (0.05). Red and pink dots represent proteins significantly increased in hepatic steatosis (*p* < 0.05 and FC > 0; *q* < 0.05 and FC > 0). Blue dots represent significantly decreased proteins (*p* < 0.05 and FC < 0). Gray dots are not significant. **B** PLS-DA plot showing separation of participants with severe hepatic steatosis (green circles) and those without steatosis (gray circles). Proteins were filtered for *p* < 0.05 and presence in ≥ 50% of samples. Ovals represent 95% confidence intervals. **C** Dot plot showing significantly enriched GO Biological Processes. Dots represent gene sets and are colored by NES; dot size reflects the number of genes in each set

We explored protein signatures associated with severe steatosis by comparing participants with severe hepatic steatosis (*n* = 43) and without hepatic steatosis (*n* = 156). Differential abundance analysis identified seven proteins significantly altered in severe hepatic steatosis after multiple testing correction, alongside an additional 63 proteins with a nominal *p* value < 0.05. Of the significantly altered proteins (*q* < 0.05), COL18A1, AFM, APOA1, INHBE, and PRG4 isoform C and F were increased, while C4A and APOA1 were decreased (Fig. 3A). PLS-DA and Hotelling’s *T*^2^ testing of significant proteins (*p* < 0.05) identified in more than 50% of samples shows significant (*p* < 0.05) separation between the two groups (Fig. 3B). These changes suggest a combined signature of enhanced extracellular matrix remodeling (COL18A1), lipid transport dysregulation (AFM), and impaired complement-mediated immunity (C4A), highlighting potential molecular pathways linked to severe hepatic steatosis. GSEA of biological processes showed significant positive enrichment for inflammation-related pathways, including regulation of humoral immune response, positive regulation of response to external stimulus, and complement activation via the alternative pathway. Lipid dysbiosis, specifically regulation of lipid biosynthetic process, was also positively enriched (Fig. 3C).

### Investigation of EV proteins associated with hepatic steatosis across subgroups

**Fig. 4:**
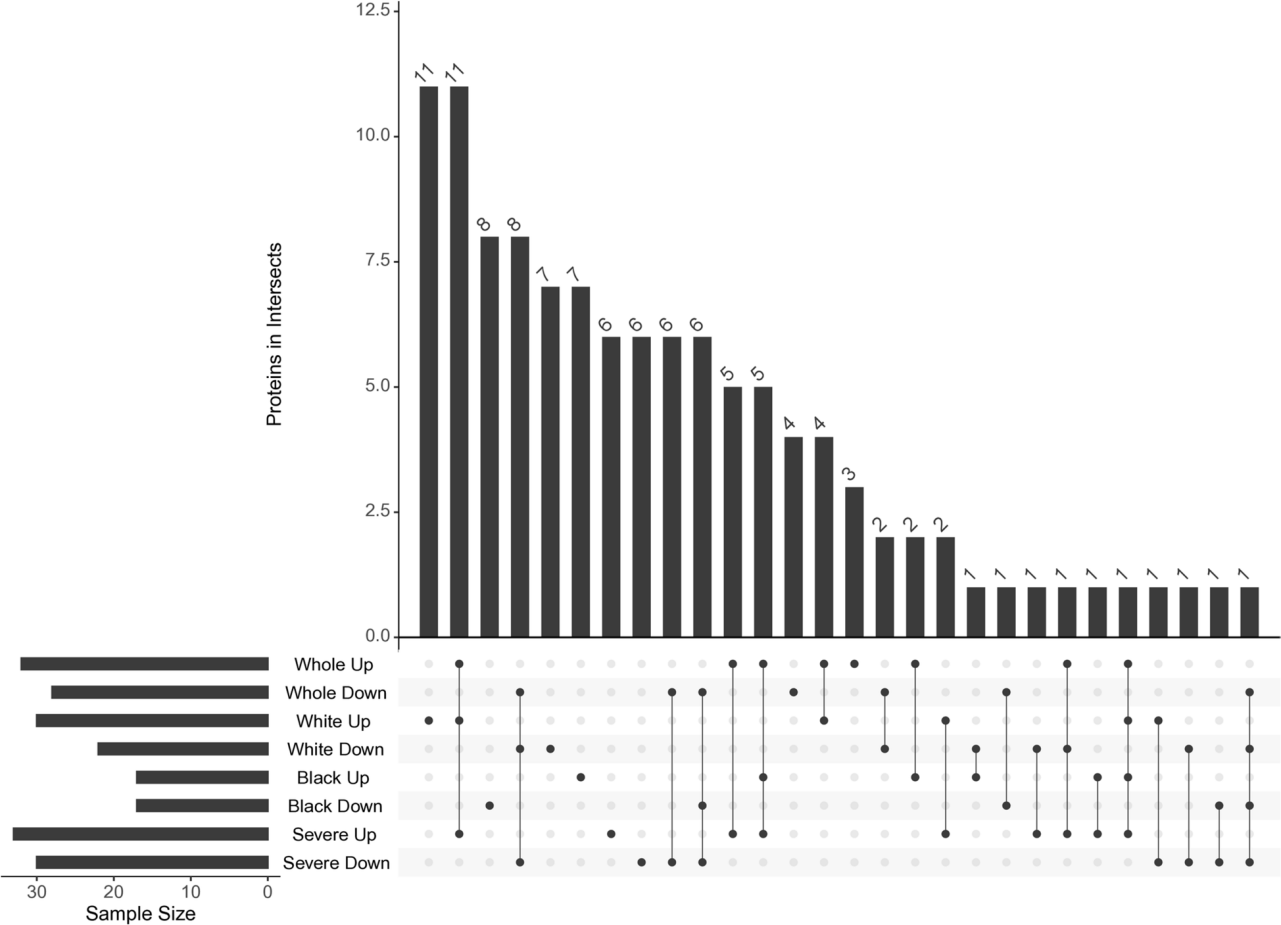
Overlap of significantly increased EV proteins across subgroups. UpSet plot displaying the intersection of proteins significantly more abundant (*p* < 0.05) in each subgroup analysis. The bottom matrix shows all possible set intersections, with filled dots indicating the sets included in each intersection and connecting lines denoting shared membership. The top bar chart shows the number of proteins in each intersection. Horizontal bars to the left indicate the total number of significant proteins in each individual subgroup. HS, hepatic steatosis

To identify EV protein signatures potentially unique to each subgroup, we compared proteins that were significantly more abundant (*p* < 0.05) across cohort-level analyses, including the overall cohort, White participants, Black participants, and those with severe hepatic steatosis (Fig. 4). INHBE emerged as the only protein consistently elevated in hepatic steatosis across all subgroup analyses (Fig. 4: Whole Up, White Up, Black Up, Severe Up). In the overall cohort (*n* = 275), three proteins—APOC2, APOA4, and IGLC6—were uniquely enriched (Fig. 4: Whole Up). Among White participants (*n* = 103), 11 proteins showed subgroup-specific enrichment: IGHA1, C1QB, C4BPA, C4BPB, LCAT, PROS1, MMRN1, SERPINA10, CFI, ANK1, and LBP (Fig. 4: White Up). In Black participants (*n* = 172), seven proteins were uniquely enriched: IGHG1, IGHG2, IGHV2-5, CFHR2, LUM, IGLV3-21, and ANTXR1 (Fig. 4: Black Up). In the severe hepatic steatosis subgroup (*n* = 43), proteins with subgroup-specific enrichment included IGKV1D-13, IGKV1-13, ICAM1, C2, C7, APOC4, and ADGRF5 (Fig. 4: Severe Up).

To explore these candidates further, we examined the expression of the encoding genes in two cohorts, one representing our previously published RNA-seq dataset (NCBI Bioproject Accession PRJNA512027; *n* = 192) [59] and the other a meta-analysis of hepatic gene expression from liver biopsy samples, integrating ten RNA-sequencing and microarray datasets, and also including PRJNA512027 (1058 samples) [60]. *INHBE* was consistently overexpressed across all three meta-analyses: hepatic steatosis vs. controls, MASH vs. controls, and MASH vs. steatosis. *AFM* also showed increased expression in each comparison, but statistical significance was only observed in the MASH vs. steatosis analysis (*Z* = 2.492; FDR = 0.0369). In contrast, *C4A* was underexpressed in all the three comparisons, though none reached statistical significance (lowest FDR = 0.145 in MASH vs. controls; Table 2). Finally, in the PRJNA512027 dataset, APOA1 and COL18A1 were significantly underexpressed in MASH relative to both controls and steatotic samples, but this pattern was not supported by the meta-analysis.

**Table 2:** Hepatic expression of genes encoding differentially abundant proteins in hepatic steatosis and MASH

Gene	HS vs. CTL				MASH vs. CTL				MASH vs. HS			
	PRJNA512027		Meta-analysis		PRJNA512027		Meta-analysis		PRJNA512027		Meta-analysis	
	*z*-score	FDR	*z*-score	FDR	*z*-score	FDR	*z*-score	FDR	*z*-score	FDR	*z*-score	FDR
*AFM*	1.971	5.95E − 01	2.074	1.74E − 01	1.819	1.49E − 01	2.076	1.21E − 01	− 0.111	9.45E − 01	**2.492**	**3.69E − 02**
*APOA1*	0.167	9.88E − 01	1.093	5.31E − 01	** − 0.515**	**1.69E − 05**	0.312	8.64E − 01	** − 5.909**	** < 1.0E − 08**	− 0.853	5.21E − 01
*C4A*	− 1.043	8.41E − 01	− 1.471	3.71E − 01	− 0.471	7.52E − 01	− 1.968	1.45E − 01	0.663	6.38E − 01	− 1.085	4.01E − 01
*COL18A1*	0.268	9.81E − 01	0.945	6.00E − 01	** − 4.606**	**7.78E − 05**	− 0.381	8.29E − 01	** − 5.711**	**4.58E − 07**	− 1.893	1.20E − 01
*INHBE*	2.676	3.49E − 01	**4.115**	**2.01E − 03**	**3.678**	**1.59E − 03**	**4.587**	**1.05E − 04**	0.388	7.94E − 01	**3.867**	**8.82E − 04**
*PRG4*	1.367	7.73E − 01	− 0.195	9.27E − 01	**4.111**	**3.99E − 04**	0.135	9.45E − 01	**3.271**	**4.69E − 03**	1.552	2.12E − 01

Gene expression data was extracted from PRJNA512027 (*n* = 192) [59] and our previously published meta-analysis of transcriptomic datasets (*n* = 1058) [60]. *HS *hepatic steatosis, *CTL *control (healthy liver), *MASH *metabolic dysfunction-associated steatohepatitis

Statistically significant results are shown in boldface

## Discussion

Current clinical guidelines often overlook sex- and age-specific differences in metabolic health, revealing a gap in targeted approaches to reduce the burden of MASLD, particularly in postmenopausal women. In this study, we profiled the circulating EV proteome in a diverse cohort of postmenopausal women and identified proteomic signatures associated with hepatic steatosis. Our findings suggest that EV cargo reflects molecular alterations relevant to liver fat accumulation in this population, supporting their potential as biomarkers for both hepatic steatosis and metabolic shifts that may precede overt liver dysfunction. Notably, we observed distinct proteomic patterns not only by steatosis status but also by race/ethnicity, indicating that EVs may capture both common and ancestry-specific disease mechanisms. This observation raises the possibility that circulating EVs integrate environmental, genetic, and metabolic influences relevant to MASLD development.

Two EV proteins, C4A and AFM, were significantly altered between women with and without hepatic steatosis. C4A was reduced, while AFM was elevated, suggesting roles in dysregulated complement activation and lipid transport, respectively, both central to MASLD pathogenesis. Elevated AFM, a vitamin E–binding hepatokine, has been linked to metabolic syndrome and hepatic inflammation [61–63]. Reduced C4A, part of the classical complement cascade, may reflect impaired immune surveillance or increased complement turnover, consistent with hepatic inflammation [34, 64, 65].

Subgroup analyses further revealed distinct EV proteomic signatures by race. In White women, proteins involved in coagulation, complement regulation, and lipid transport, including complement C4 binding protein alpha (C4BPA), lecithin-cholesterol acyltransferase (LCAT), protein S (PROS1), complement C4 binding protein beta (C4BPB), and multimerin 1 (MMRN1), were elevated in hepatic steatosis. These proteins implicate dysregulation of innate immunity and lipoprotein remodeling and warrant further study to clarify their specific contributions. In contrast, Black women exhibited enrichment of immunoglobulin-related proteins (e.g., IGHG1, IGHG2, IGHV2-5, IGLV3-21), suggesting differential activation of humoral immune response. Additional subgroup-specific proteins such as complement factor H-related protein 2 (CFHR2), thrombospondin 4 (THBS4), lumican (LUM), and anthrax toxin receptor 1 (ANTXR1) suggest potential ancestry-associated differences in inflammatory and matrix remodeling pathways [66–68]. However, these results require cautious interpretation as self-reported race/ethnicity, while a useful proxy, does not fully capture genetic ancestry or account for its continuous variation and substructure across population groups [69].

In participants with severe hepatic steatosis, EV protein signatures were enriched for markers of inflammation, complement activation, and dysregulated lipid metabolism. Among these, INHBE, COL18A1, AFM, and PRG4 were significantly elevated, while C4A and APOA1 were reduced. Enrichment of pathways involved in humoral immune responses and lipid biosynthesis further supports the hypothesis that these EV changes reflect worsening metabolic and immune dysregulation in advanced steatosis. These findings suggest that EV profiling may not only detect early disease but also stratify severity and capture pathophysiologic processes driving progression toward MASH.

While the EV biomarker literature in MASLD remains limited, several studies have reported EV-derived proteins associated with liver pathology. For example, Sakane et al. [70] identified differential abundance of proteins, including fibulin-3 and gelsolin, in EVs from patients with advanced fibrosis, while Geng et al. [71] observed elevation of hepatocyte-associated proteins such as epithelial cell adhesion molecule (EpCAM) and ITGA3 in EVs from precision-cut liver slices of patients with cirrhosis, with upregulated proteins enriched in complement and coagulation cascades and downregulated proteins associated with xenobiotic and drug metabolism pathways. Our previous work in adolescents with hepatic steatosis also detected EV-derived proteins involved in complement activation and lipid metabolism, several of which exhibit high hepatic expression (e.g., FGL1, RBP4, GC) [34]. Collectively, these studies reinforce the potential of EV proteomics to reflect liver biology and disease progression, and our current findings extend this work by identifying proteins, including AFM, INHBE, COL18A1, and PRG4, that may serve as candidate hepatocyte-derived biomarkers of steatosis in postmenopausal women.

To determine whether EV protein cargo changes reflect underlying hepatic gene expression, we analyzed transcript levels of the corresponding genes in two liver transcriptomic datasets comprising over 1000 samples. INHBE was significantly overexpressed in both hepatic steatosis and MASH compared to controls, and in MASH compared to steatosis, consistent with a potential role in disease progression. AFM was significantly upregulated in MASH compared to hepatic steatosis, with similar but nonsignificant trends in both MASH and hepatic steatosis relative to controls, suggesting its involvement in later stages of disease. C4A expression was reduced in MASH compared to both control and steatotic livers, in accordance with the observed decrease in EV protein levels and indicative of a potential loss of complement-mediated protective mechanisms in advanced disease. APOA1 and COL18A1 were significantly underexpressed in MASH in the PRJNA512027 dataset though these differences were not observed in the meta-analysis. Together, these findings suggest that the EV proteins identified in serum may reflect, at least in part, underlying hepatic transcriptional alterations.

Although EV protein abundance does not consistently parallel hepatic gene expression, some convergence becomes evident in advanced disease. This partial overlap may reflect posttranscriptional regulation, differences in protein turnover or secretion, or contributions from non-parenchymal cell types to the EV proteome. Accordingly, EVs may provide complementary insights into disease biology, particularly when transcriptomic alterations are subtle or heterogeneous.

Of the EV proteins identified in our analyses, INHBE was the most consistently elevated across cohort and subgroup analyses, and its transcriptional upregulation was observed across two different liver datasets. Although classical inhibins A and B decline with menopause due to loss of ovarian function [72], INHBE encodes a distinct β-subunit of activin E, a hepatokine increasingly recognized for its role in metabolic regulation. Recent studies have shown that *INHBE* expression is induced by hepatic steatosis and insulin resistance [73–75], consistent with our finding of elevated EV-derived INHBE across all subgroups. These data suggest that INHBE upregulation in hepatic steatosis may represent a liver-derived compensatory response to metabolic stress rather than a reflection of altered gonadal hormone signaling. In this context, INHBE may serve as a relevant biomarker of liver–endocrine crosstalk in postmenopausal women with MASLD.

Our findings extend the emerging literature supporting circulating EVs as potential biomarkers of liver and metabolic disease. The observation that EV proteomes capture both common and ancestry-specific signatures of hepatic steatosis highlights their potential for precision phenotyping. At the same time, specificity remains an important consideration: EV cargo is influenced by multiple tissues and systemic conditions and overlap with other metabolic or inflammatory states may limit disease discrimination. Thus, while the current study supports the value of EV profiling, further work integrating longitudinal follow-up, complementary omics, and rigorous validation cohorts will be needed to establish the clinical utility of EVs in MASLD.

Despite several strengths, including the use of a well-characterized, community-based cohort and focus on a high-risk population, several limitations should be noted. First, hepatic steatosis was assessed via ultrasound, an imaging modality with limited sensitivity for mild fat accumulation and lacking quantitative resolution [20–23]; histology and non-invasive fibrosis staging were not available for this cohort. Similarly, our analyses focused on steatosis, the earliest detectable lesion in MASLD, whereas fibrosis and inflammation are stronger predictors of clinical outcomes. Studies incorporating histologic or advanced imaging phenotyping and more advanced disease will be valuable for validating and extending our results. Second, while our sample size is larger than many prior proteomic studies in MASLD [76, 77], it may have limited our ability to detect subtle differences or perform additional subgroup analyses. Larger, multi-center cohorts are needed to confirm ancestry-specific EV signatures and to explore the influence of comorbidities such as obesity, diabetes, or hormone therapy on EV proteomic profiles. Third, the single-site, cross-sectional design constrains generalizability and precludes inference about causality or temporal dynamics of EV changes. Fourth, we could not directly assess the tissue of origin of circulating EV proteins. While some identified proteins are liver-enriched (e.g., AFM and INHBE), others may reflect contributions from immune or adipose tissue. EVs were also isolated from serum, and co-isolation of lipoproteins such as HDL cannot be fully excluded; although canonical EV markers were enriched, lipoprotein-associated proteins may remain a technical limitation. Finally, while our study integrates transcriptomic data from two liver datasets to strengthen biological interpretation, we acknowledge the absence of an independent validation cohort. Therefore, our findings should be considered exploratory, and further work integrating longitudinal follow-up, complementary omics, and rigorous validation cohorts will be required to establish the clinical utility of EVs in MASLD.

## Conclusions

This study demonstrates that circulating EV proteomes differ by hepatic steatosis status in postmenopausal women and reveal both shared and ancestry-specific molecular patterns. While these findings suggest that EVs may capture systemic metabolic perturbations relevant to MASLD pathogenesis, they should be interpreted as exploratory. Candidate EV proteins identified here warrant further evaluation in independent, well-characterized cohorts to confirm their potential as biomarkers or mechanistic mediators. Future research should prioritize longitudinal, multi-omic studies with rigorous phenotyping to establish whether EV profiles can improve risk stratification and provide insight into the biology of MASLD progression.

## Supplementary Information

Additional file 1. Fig. S1 shows the results of principal component analyses and Table S1 presents characteristics of participants with hepatic steatosis, by diabetes status.

## Data Availability

The mass spectrometry proteomics data have been deposited to the ProteomeXchange Consortium via the PRIDE [78] partner repository. SWAN data are archived at https://www.icpsr.umich.edu/web/ICPSR/series/00253 and the Aging Research Biobank [https://agingresearchbiobank.nia.nih.gov/studies/swan/?search\_term=SWAN](https:/agingresearchbiobank.nia.nih.gov/studies/swan/?search_term=SWAN). The Michigan site- specific liver datasets used and/or analyzed during the current study are available from author Carrie Karvonen-Gutierrez ([ckarvonne@umich.edu](mailto:ckarvonne@umich.edu)) on reasonable request.

## Abbreviations

AFM: Afamin
ANTXR1: Anthrax toxin receptor 1
ANCOVA: Analysis of covariance
ANK1: Ankyrin 1
APOA1: Apolipoprotein A1
APOA4: Apolipoprotein A4
APOC2: Apolipoprotein C2
APOC4: Apolipoprotein C4
BMI: Body mass index
C2: Complement component 2
C4A: Complement component 4A
C4BPA: Complement component 4 binding protein alpha
C4BPB: Complement component 4 binding protein beta
C7: Complement component 7
CFHR2: Complement factor H-related protein 2
CFI: Complement factor I
DDA: Data-dependent acquisition
DIA: Data-independent acquisition
DAPs: Differentially abundant proteins
T2D: Diabetes mellitus
EVs: Extracellular vesicles
FDR: False discovery rate
GOBP: Gene Ontology Biological Processes
GSEA: Gene Set Enrichment Analysis
HbA1c: Hemoglobin A1c
HPLC: High-performance liquid chromatography
IGHA1: Immunoglobulin A1
IGHG1: Immunoglobulin heavy constant gamma 1
IGHG2: Immunoglobulin heavy constant gamma 2
IGHV2-5: Immunoglobulin heavy variable 2–5
IGKV1-13: Immunoglobulin kappa variable 1–13
IGKV1D-13: Immunoglobulin kappa variable 1D-13
IGLC6: Immunoglobulin lambda constant 6
IGLV3-21: Immunoglobulin lambda variable 3–21
INHBE: Inhibin beta E
IRB: Institutional Review Board
iRT: Integrated retention time
ICAM1: Intercellular adhesion molecule 1
LCAT: Lecithin-cholesterol acyltransferase
LBP: Lipopolysaccharide binding protein
LC: Liquid chromatography
LC-MS: Liquid chromatography–mass spectrometry
LUM: Lumican
MS: Mass spectrometry
MASH: Metabolic dysfunction-associated steatohepatitis
MASLD: Metabolic dysfunction-associated steatotic liver disease
MI-SWAN: Michigan site of the Study of Women’s Health Across the Nation
MMRN1: Multimerin 1
PLS-DA: Partial least squares discriminant analysis
PASEF: Parallel accumulation serial fragmentation
PBS: Phosphate buffered saline
PC: Principal component
PCA: Principal component analysis
PROS1: Protein S
PSMs: Proteome spectral matches
SERPINA10: Serpin family A member 10
SEC: Size exclusion chromatography
SWAN: Study of Women’s Health Across the Nation
T2D: Type 2 diabetes
TG: Triglyceride
VSN: Variance stabilization normalization
