# Supplementary material for: Hepatic steatosis in postmenopausal women is characterized by distinct serum extracellular vesicle proteomic signatures
Source: BMC Med. 2025 Dec 7;24:24. doi: 10.1186/s12916-025-04571-4 (PMC12797421; doi:10.1186/s12916-025-04571-4)
Supplement: Supplementary file 1 — Additional file 1. Fig. S1 shows the results of principal component analyses and Table S1 presents characteristics of participants with hepatic steatosis, by diabetes status. [file 12916_2025_4571_MOESM1_ESM.docx]

**Fig. S1.** **Correlation of Protein Abundance to Clinical Variables. (A)** Scree plot showing percent explained variance (y-axis) in each principal component (x-axis). Seventy-five percent of explained variation is contained within the first 24 principal components. (**B)** Principal component Spearman clinical correlation plot shows significance of correlation of protein abundances to clinical variables. **(C)** Principal component Spearman $r^{2}$clinical correlation plot shows significance of correlation of protein abundances to clinical variables. Asterisks in cells for panels C and D represent clinical variables with significant correlation to proteomics data at given PC.


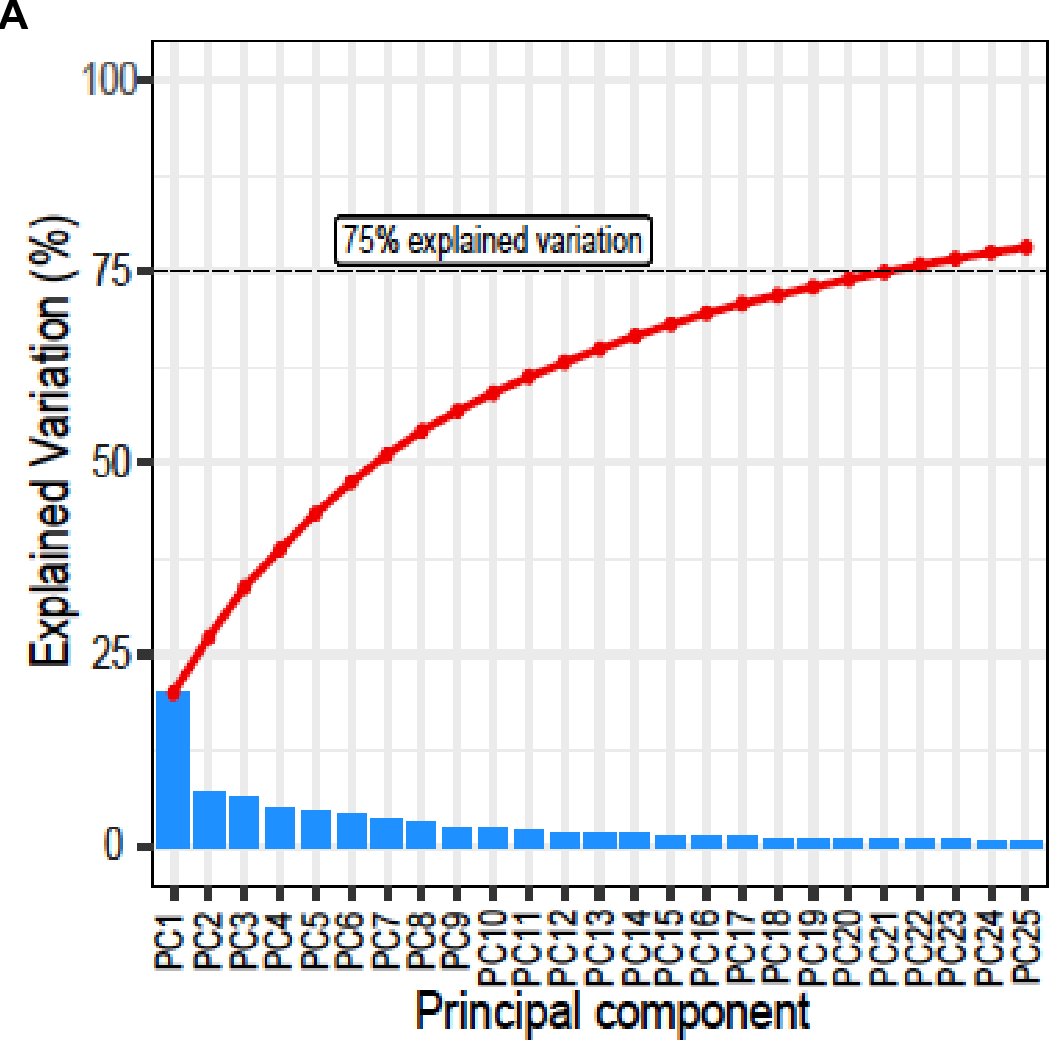

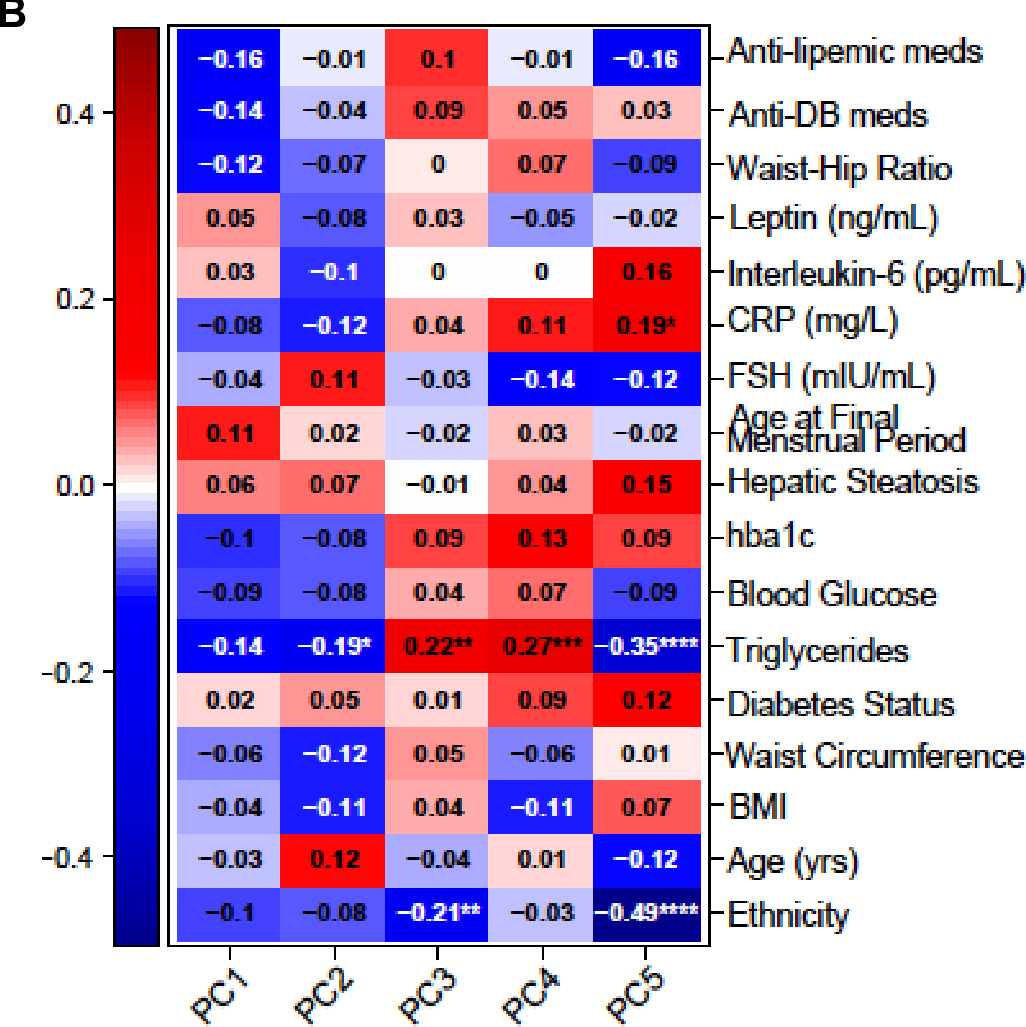


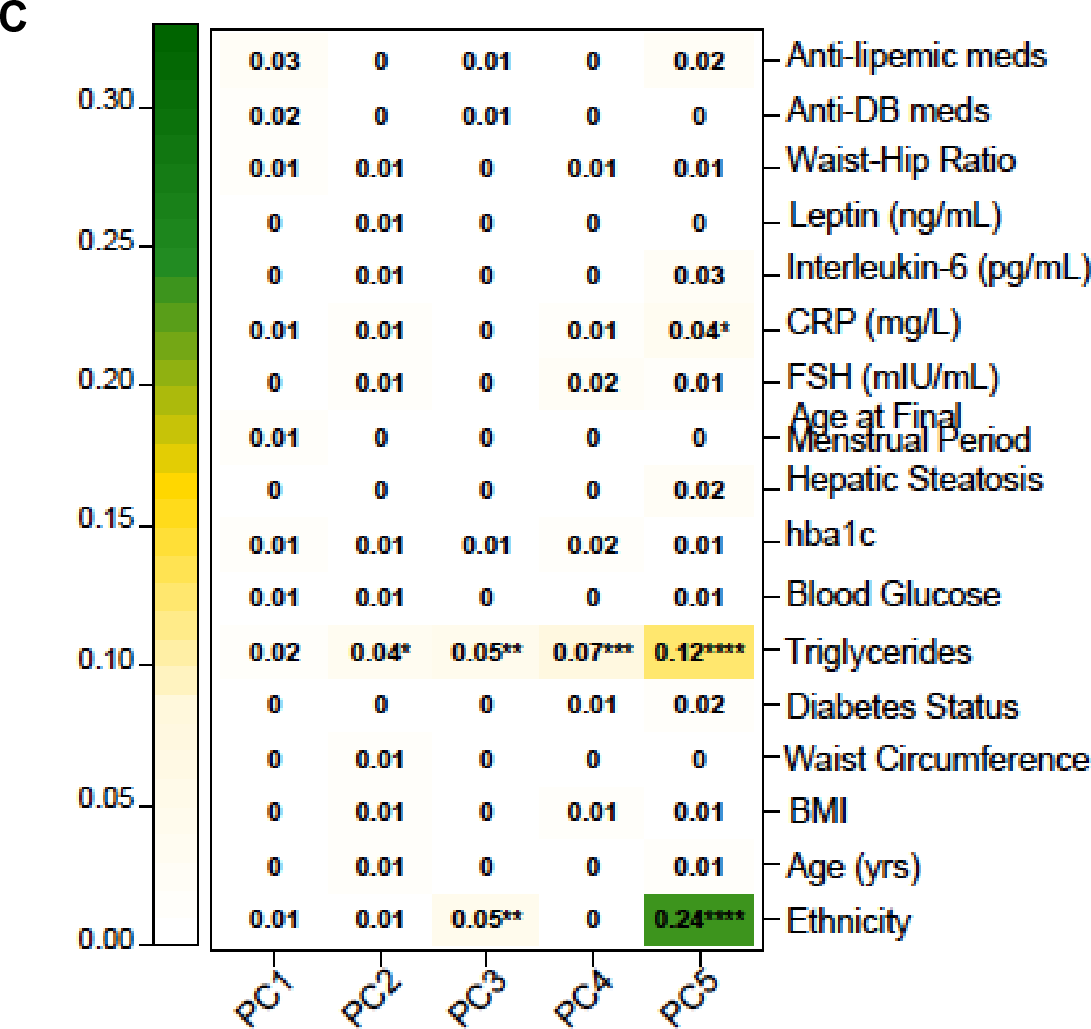


| **Table S1. Study cohort demographic information and clinical characteristics among women with hepatic steatosis, by diabetes status (N = 75):** | | |
| --- | --- | --- |
| **Parameter** | **No Diabetes** | **Diabetes** |
| **N** | 43 | 32 |
| **Age (y)** | 59.84 ± 3.04 | 59.00 ± 2.64 |
| **Race n (%)** |  |  |
| Black | 24 (55.81) | 14 (43.75) |
| White | 19 (44.19) | 18 (56.25) |
| **BMI (kg/m^2^)** | 36.50 ± 7.66 | 37.91 ± 7.58 |
| **WC (cm)** | 106.30 ± 13.68 | 114.18 ± 10.98 |
| **TG (mg/dL)** | 120.49 ± 72.53 | 198.59 ± 94.38 |
| **FG (mg/dL)** | 98.12 ± 13.22 | 168.31 ± 103.27 |
| **HbA1c (%)** | 5.76 ± 0.50 | 8.07 ± 2.03 |
